# Adherence to the WHO surgical safety checklist: an observational study in a Swiss academic center

**DOI:** 10.1186/s13037-019-0194-4

**Published:** 2019-03-12

**Authors:** René Schwendimann, Catherine Blatter, Marc Lüthy, Giulia Mohr, Thierry Girard, Siegfried Batzer, Erica Davis, Henry Hoffmann

**Affiliations:** 1 Patient Safety Office, University Hospital Basel, Spitalstrasse 22, 4031 Basel, Switzerland; 2 Department Public Health DPH, Nursing Science, Faculty of Medicine, University of Basel; Bernoullistrasse 28, 4056 Basel, Switzerland; 3 Department of Anesthesiology, University Hospital Basel, Spitalstrasse 21, 4031 Basel, Switzerland; 4 Department of Surgery, University Hospital Basel, Spitalstrasse 21, 4031 Basel, Switzerland

**Keywords:** Surgical checklist, Team time out, Team sign out, Adherence, Short report

## Abstract

**Background:**

The World Health Organization (WHO) Surgical Safety Checklist is used globally to ensure patient safety during surgery. Two years after its implementation in the University Hospital Basel’s operating rooms, adherence to the protocol was evaluated.

**Methods:**

This mixed method observational study took place in the surgical department of the University Hospital of Basel, Switzerland from April to August 2017. Data collection was via individual structured interviews with selected OR team members regarding checklist adherence and on-site non-participant observations of Team Time Out and Team Sign Out sequences in the OR. Data were subjected to thematic analysis and descriptive statistics compiled.

**Results:**

Comprehensive local expert interviews indicated that individual, procedural and contextual variables influenced the application of the checklist. Facilitating factors included well-informed specialists who advocated the use of the Checklist, as well as teams focused on the checklist’s intended process and on its content. In contrast, factors such as staff insecurity, a generally negative attitude towards the checklist, a lack of teamwork, and hesitance to complete the checklist, hindered its implementation.

The checklist’s application was evaluated in 104 on-site observations comprising of 72 Team Time Out (TTO) and 32 Team Sign Out (TSO) sections. Adherence to the protocol ranged between 96 and 100% in TTO and 22% in TSO respectively. Lack of implementation of the TSO was mainly due to the absence of one of the key OR team members, who were busy with other tasks or no longer present in the operating room.

**Conclusion:**

The study illustrates factors, which foster and hinder consistent application of the WHO surgical safety checklist namely individual, procedural and contextual. It also demonstrates that the TTO was consistently and correctly applied, while the unavailability of key OR team members at sign-out time was the most common reason for omission or incomplete use of the TSO.

## Background

The *“WHO Surgical Safety Checklist”* is used globally to ensure patient safety during surgery [1] and has demonstrated potential to be effective at reducing surgical complication and mortality rates [2–4]. The WHO checklist improves ‘patient safety and inter-discipline communication’ and prevents ‘avoidable complications by emphasising current safety procedures [5]. Despite widespread adoption, surgical “never events” and other OR related serious incidents still occur, which could be due to problems regarding compliance to the checklist [6]. To successfully implement the checklist, it is imperative to have key team members in a supervisorial role. This facilitates team interaction regarding adjustment of checklists, and consideration of local contextual factors [4]. Adopting a stakeholder-driven approach while engaging all OR personnel (including surgeons, anesthesiologists, nurses, and technical staff) in a multifaceted intervention can significantly increase surgical safety checklist adherence [7].

### Local use of the WHO surgical safety checklist

In 2014 the WHO surgical checklist was introduced to the Department of Surgery at the University Hospital of Basel (USB), in Basel, Switzerland [8]. We modified this checklist by focusing on what we considered the two most important points, the two step process “Team Time Out” (TTO) and Team Sign Out (TSO) which was executed in the OR with an already anaesthetised patient.

The TTO takes place before the first incision and involves the entire OR team. It is initiated by the surgeon and includes an introduction round of OR team members, reconfirmation of the patient’s identity, along with remarks/special information regarding the anaesthesia process, and the operation itself. Only after this step may the operation be started. Before the final suturing and exiting of the OR, the surgeon initiates the TSO. Ensuring that, after the operation’s completion, all essential steps have been taken, such as counting instruments and swabs to confirm that no foreign objects have been inadvertently left inside the patient.

In spite of initial positive experiences after the checklist’s implementation in the hospital’s surgical departments, the long-term adherence using the checklist remained unclear. Therefore in 2017, it was decided that the checklist would be examined with a systematic follow-up evaluation of TTO and TSO application in. The focus of this evaluation were the following two questions: “Which factors foster or hinder the consistent application of the checklist?” and “Is the checklist consistently and correctly applied during surgical procedures?”

## Methods

This prospective observational study took place in the surgical department of the University Hospital of Basel, Switzerland from April to August 2017. The data collection procedure included individual structured interviews with selected local OR team members regarding checklist adherence, as well as on-site non-participant reports regarding the target checklist procedures.

**Fig. 1:**
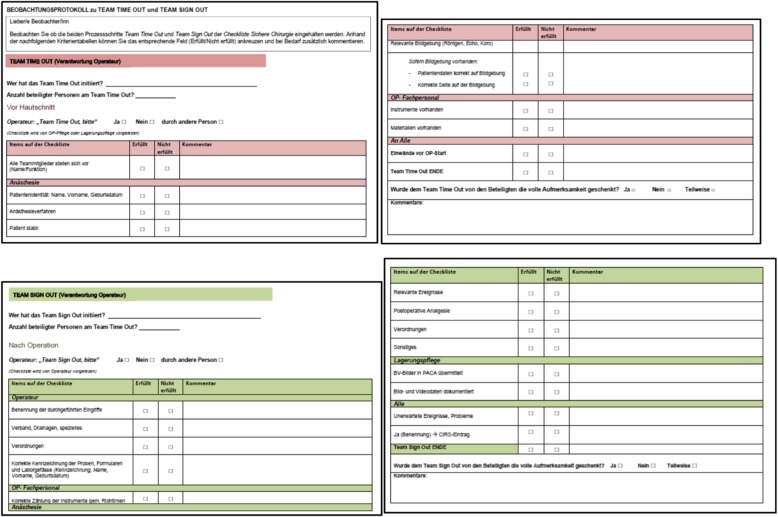
Excerpts from the original observer reports regarding TTO and TSO checklist items

The interviews with OR team members were conducted by one researcher (RS) and included open-ended questions on what fosters and hinders OR professionals’ adherence to the checklist. Interviewees’ statements were audio-taped and transcribed verbatim. The resulting transcripts were subsequently subjected to thematic analysis [9]. The on-site observations were performed by two trained expert non-OR staff members (SB, GM) in a convenience sample of various types of ORs during TTO and TSO sequences. The observation form to be completed consisted of the TTO and TSO checklist items (Fig. 1). Observation data on consistency and correctness of TTO and TSO were analysed descriptively using Microsoft Excel standard software.

## Results

Eleven experts from the OR were interviewed including six surgeons and anaesthesiologists as well as five OR and nurse anaesthetists. The average interviewee age was 46.7 years, with OR experience averaging 16.5 years. The interview results were thematically grouped according to participants’ core statements.

### Fostering factors regarding checklist implementation

Interviewees’ statements regarding checklist implementation and application specified individual, procedural, and contextual factors. Individual factors included personal attitude and approach. This was expressed in terms of “being well-informed and committed” and “standing behind” the checklist concept, as well as in relation to application of the checklist application, e.g., “considering it important” and “believing in it’s philosophy”.

The procedural factors influencing the checklist’s application were highly focussed on “regularity of…[checklist] application” and “procedure safety”, the “moment of pause before the procedure” when “everyone is focused”, “the checklist points are read up on explicitly” the essential elements, e.g., “diagnosis, problems, risks and procedures” are in sharp focus and “no-one is busy with other tasks”.

Contextual factors such as the work environment and collaboration between expert staff also played an important role. “Quiet in the OR” and “an atmosphere which allows everybody to participate during the TTO during these two to three minutes” were considered as particularly important factors.

### Factors hindering implementation of the checklist

The execution of the checklist was also hindered by individual, procedural and contextual factors. Individual barriers to its application include negative attitudes, insecurity and resistance of OR professionals. For example, “inertia of people regarding change from their established routine (from the pre-checklist period)”, “hybris”, “lack of self-discipline”, “not listening carefully”, as well as “lack of insight and acceptance as to the meaning and purpose” of the checklist were mentioned. Procedural factors hindering the checklist’s application included disruptions in information flow, lack of teamwork, and the effort involved in its execution. On this topic, experts noted that additional barriers included “being unclear regarding to whom the questions are posed”, “the checklist being crammed in”,“persons arriving too late” or that “people conversed during the TTO”. In addition, “it’s length” with “unnecessary and irrelevant topics, if in a hurry” was cited as a barrier. As to the contextual factors, these concerned work and environmental conditions such as distractions, interruptions and time pressure. Also named were OR background noise, e.g., “a high mechanical noise level” or “telephones ringing”.

### Observations of team time out and team sign out

To evaluate if the surgical checklist was consistently and correctly applied at our institution two specially trained researchers conducted a total of 104 onsite observations. These included 72 TTO and 32 TSO sequences across all surgical disciplines to which the “General” checklist applies. The median numbers of experts present in the OR for TTO and TSO were 7 (range: 3–12) and 5 (range: 2–6), respectively. In 22 observed situations (21%), the presence of the observers was explicitly acknowledged by members of the OR team.

The 72 observed TTO sequences were initiated mainly either by the surgeons (58%) or the OR nursing staff (34%). In 87% of observations the checklist was read entirely as per protocol. For TTO, in accordance with the checklist protocol, the 21 relevant checklist items were read by members of the OR team, and were almost completely (96–100%) fulfilled (see Table 1). Here, only 75% of observation data forms include positive responses to the prompt: “Surgeon: Team Time Out, please,” because the requests were made not by surgeons but by other members of the OR team (21%) or omitted (4%).

**Table 1:** Team time out items (*n* = 72 direct observations)

Team Time Out (TTO) before skin incision	Item confirmed % (n)
Operating surgeon: «Team Time Out, please»	75 (54)
All team members have introduced themselves by name and role	99 (71)
*Anesthetist*	
Patient identification: Family name, first name, birth date	100 (72)
Type of anesthetic procedure	100 (72)
Patient is stable	99 (71)
Allergies	97 (70)
Antibiotic prophylaxis	97 (70)
Specific perioperative risks	99 (71)
Any patient-specific concerns and precautions	99 (71)
*Operating surgeon*	
Type of surgery and marked site	99 (71)
Specific risks	99 (71)
Intraoperative medications	99 (71)
Duration of surgery	100 (72)
Anticipated blood loss	99 (71)
Correct patient positioning	99 (71)
Specific equipment (e.g. microscope)	99 (71)
Essential imaging (X-ray, echo, coronary etc.)	97 (70)
*OR-Nurse*	
Equipment available	97 (70)
Supplies available	96 (69)
*Whole OR team*	
Any objections before OR	99 (71
Team Time Out «completed»	99 (71)

Of the 32 observed TSO sequences, only 7 (22%) were correctly initiated and applied. In general, the TSO items were not fully executed (see Table 2). In all but 7 cases, non-application of the TSO was blamed on the absence of the surgeon or the OR nurses, who had already left the OR, or of the anaesthesiologists, who were engaged elsewhere (e.g. caring for recovering patients).

**Table 2:** Team sign out items (*n* = 32 direct observations)

Team Sign Out (TSO) before patient leaves OR	Item confirmed % (n)
Operating surgeon: «Team Sign Out, please»	22 (7)
*Operating surgeon*	
Name of the procedure	22 (7)
Dressing, Drainage, Specials	22 (7)
Prescriptions	19 (6)
Correct specimen labelling, forms and laboratory containers (Patients name and birth date)	16 (5)
*OR-Nurses*	
Completion of instrument, sponge and needle count	16 (5)
Image intensifier images transmitted to PACS^a^	13 (4)
Images- and Video data saved	13 (4)
*Anesthetist*	
Relevant events	19 (6)
Postoperative Analgesia	19 (6)
Prescriptions	19 (6)
Other	16 (5
*Whole OR team*	
Unexpected, critical events	16 (5)
If yes, specified and reported (CIRS)^b^	13 (4)
*Team Sign Out* «completed»	19 (6)

^a^Picture archiving and communication system

^b^Critical incident reporting system

## Discussion

Our study identified individual, procedural and contextual factors, which supported or limited the application of the surgical checklist. It demonstrated that the TTO portion of the checklist was consistently and correctly applied, while the TSO was generally lacking in full participation mainly due to the absence of key OR team members, who were engaged otherwise at that time point or no longer present in the operating room.

Factors that encouraged adherence to the checklist was the feeling of being part of a team, a shared goal of patient safety and full staff participation. Factors that reduced adherence were feelings of insecurity, certain team members’ resistance to checklist implementation and key team members not being present or fully involved. Although surgeons are commonly described as being the leaders in the OR, another study revealed similar observations i.e. the staff surgeon was often not in the OR during the briefing and the process was delegated to another OR team member [10]. Furthermore, the most common barrier to checklist implementation was active resistance or passive noncompliance from individuals in the OR team, most frequently from senior surgeons and/or anesthesiologists [11]. Other studies demonstrate that introducing a surgical safety checklist requires modification in the workflow of the surgical staff resulting in an increased workload [12]. On the other hand, increased workload can result in decrease utilization of a checklist, but research has also shown that team players working with the WHO-surgical checklist attained substantially higher levels of awareness about the benefits of the checklist than those who did not implement it [13].

Non-adherence with surgical checklists included inadequate communication and the absence of committed key members of the OR team, which lead to incompletion of the surgical checklist [11, 14], for instance, the TSO or hindering factors expressed by the experts.

Lasting implementation of the checklist hinges on clear and concise communication regarding both its purpose and its application. Along with its practical execution, the checklist’s application refers to its introduction and process of long-term implementation. This includes coaching if necessary, and consistent specific education and training; [15] all vital elements to make the checklist effective [16]. Moreover, there are new ways to support its use, such as delivering the checklist via audio. Which enables fully addressing items for both team time-out and sign-out as well as improvement in overall team participation at time-out [6]. Although the USB’s department of surgery initially advocated the introduction of the WHO checklist for safe surgery, gaps in its daily use persist. Individual OR teams’ TSO non-adherence might reflect the ability of each surgical discipline’s representative to sustainably enforce the accepted policy. Indeed, compliance with individual elements of the checklist might vary by surgical specialties [17]. In another study, the sign-out was also not fully integrated into existing OR procedures. Difficulties included identifying an appropriate time for the checks; if left until after closing, the operating surgeon was often no longer present, but if completed earlier, the nurses’ final counts were often not yet complete [18].

In order to strengthen future TSO use, the checklist procedure has to be adapted to allow improvement of crucial workflow points regarding patient safety (e.g., addressing concerns regarding patient recovery and management). Supported by key OR team members, firm directives from chief surgeons would promote and facilitate checklist adherence by demonstrating leadership engagement [19].

Strengths of this study include the thematic analysis of the expert’s in-depth interviews, which provided individual, procedural and contextual feedback that brought about a necessary revision to the checklist. Limitations include sample bias, which might not fully cover other surgical team’s checklist needs across various surgical disciplines.

## Conclusions

This study reveals that TTO was consistently applied while only one-fifth of TSO was conducted, which indicates a need for future action. The expert interviews displayed that the application of the checklist is both fostered and hindered by individual, procedural and contextual factors. In addition to supporting the successful contextual adaptation of the checklist, consistent implementation will require additional training at the levels of both the individual medical professionals and the OR teams. Consistent use of the Checklist remains vital to patient safety in surgery in which role models are of particular importance besides formal training efforts to foster a culture of safer OR practices.

## Abbreviations

CIRS: Critical incident reporting system
OR: Operating Room
PACS: Picture archiving and communication system
TSO: Team sign out
TTO: Team time out
